# Moving towards an Understanding of the Role of the Inferior Fronto-Occipital Fasciculus in Language Processing

**DOI:** 10.3390/neurosci5010003

**Published:** 2024-01-02

**Authors:** Princess Eze, Efrem Omorotionmwan, Jacqueline Cummine

**Affiliations:** 1 Neuroscience and Mental Health Institute, University of Alberta, Edmonton, AB T6A 2G4, Canada; poeze@ualberta.ca (P.E.);; 2 Communication Sciences and Disorders, University of Alberta, Edmonton, AB T6A2 G4, Canada

**Keywords:** inferior fronto-occipital fasciculus, language, dorsal pathway, ventral pathway

## Abstract

Evidence has been provided for a clear structural distinction between the dorsal and ventral portions of the inferior frontal occipital fasciculus (IFOF). As such, there is reason to propose that there might also be a functional differentiation of the dorsal and ventral components of the IFOF. Here, we explored three main hypotheses/schools of thought with regards to the functional frameworks of the dorsal and ventral components of the IFOF: (1) the phonological vs. semantic processing hypothesis, (2) the difficult vs. non-difficult task processing hypothesis and (3) the automatic vs. non-automatic processing hypothesis. Methods: Participants (N = 32) completed a series of behavioral tasks that aligned with each of the main hypotheses. Using a regression-based approach, we assessed the unique contribution of behavioral performance to dorsal and ventral IFOF white matter indicators (i.e., fractional anisotropy and mean diffusivity). Results: We found significant relationships between ventral IFOF indices and orthographic awareness (*p* = 0.018) and accuracy (*p* = 0.009). Overall, our results provide converging evidence that the IFOF primarily operates as a ventral language tract in adults. Thus, the structural distinction between dorsal and ventral IFOF does not manifest as a parallel functional distinction.

## 1. Introduction

As modern imaging methods become more advanced, there have been new insights about the functional and structural networks present in high-level processes like language. A key part of these neural networks is the underlying white matter circuitry that connects essential brain regions involved in language [1]. It is known that white matter tracts allow communication between different brain regions; therefore, it is likely that these tracts are needed for language processes. Many studies have sought to decipher how complex processes like speech production, speech perception, and literacy are related to underlying brain connectivity [2,3,4,5,6,7,8]. Several of these studies have identified networks of interconnected brain regions involved in these language processes. As the field continues to expand, there is a growing consensus that language processes are differentially supported via ventral (e.g., uncinate fasciculus) and dorsal (e.g., arcuate fasciculus) white matter pathways [6]. However, the role of tracts that are more medially located or that have projections into both dorsal and ventral regions, such as the inferior fronto-occipital fasciculus (IFOF), remains uncertain.

### 1.1. Diffusion Tensor Imaging and White Matter Tractography

Diffusion Tensor Magnetic Resonance Imaging (DTI) is an in vivo technique used to study white matter in the human brain [9,10]. The diffusion of water in the brain matter tends to be very random, and this is termed isotropic diffusion. However, in the axons, structural barriers keep water following in the direction of the tracts, and this is termed anisotropic diffusion. DTI can be used to map axonal connections between brain regions by measuring the diffusion of water molecules in the brain tissue [9,10]. DTI uses eigenvalues that represent the magnitude of diffusion along the orientations of the eigenvectors, which are the three principal axes of the ellipsoid (x-axis, y-axis, z-axis) [11]. The main assessments used with DTI are obtained from these eigenvalues. These assessments include fractional anisotropy (FA) and mean diffusivity (MD). FA is calculated by obtaining the ratio of the standard deviation and root mean square of the eigenvalues. The value of FA ranges between 0 (diffusion in all directions) and 1 (highly restricted diffusion). For example, in the cerebral spinal fluid (CSF), where diffusion is the same in all directions, FA is close to 0, but in organized white matter bundles like the corpus callosum, FA is closer to 1. MD is calculated by dividing the sum of the eigenvalues by 3 (i.e., the average). MD is a measure of how freely the molecules can move in all directions, so MD is generally less precise than FA. Both variables are indirect measures of the microstructure of white matter, and correlations between behavioral tasks and the integrity of these white matter structures serve to inform us about language processing in the brain [11].

### 1.2. Structural Framework of the Inferior Frontal Occipital Fasciculus

Multiple studies have indicated that there is a clear structural distinction between the dorsal and ventral portions of the inferior frontal occipital fasciculus (IFOF). Martino et al. [12] were the first to identify a differentiation of the IFOF into its dorsal and ventral components. They conducted a post-mortem study, which led to the discovery that the IFOF could be separated at the ventral portion of the external capsule. They concluded that the superficial dorsal component connects the frontal operculum to the superior parietal lobule (SPL) and superior/middle occipital gyri. They also noted that the deep ventral component projected to the inferior temporal gyrus, the temporal-occipital sulcus, and the inferior occipital gyrus. The post-mortem findings of Martino et al. [12] have been replicated in diffusion tensor tractography studies, which provides more support for the existence of dorsal and ventral IFOF components. Their findings were supported by Hau et al. [13] and Wu et al. [14], who conducted in vivo tractography studies and found that the dorsal IFOF projected to the SPL in the majority of their participants. Caverzasi et al. [15] used a technique called q-ball reconstruction and identified that the dorsal IFOF projected to the SPL in all their participants. Caverzasi et al. [15] also identified frontal cortical connections of IFOF directed near superior and middle frontal gyri. Sarubbo et al. [16] conducted anatomical and DTI studies that support the findings of a dorsal and ventral IFOF. Their anatomical results supported the existence of a superficial dorsal segment, and they identified posterior, middle, and anterior portions of the deeper ventral component that all connect to the frontal areas of the cortex. Notably, they observed the same frontal connections to the middle frontal gyrus that Caverzasi et al. [15] identified. Additionally, their selections of ROIs during their tractography analysis did not include any portions of the parietal lobe, so they failed to see the connections of the dorsal IFOF to the SPL, as seen in the other studies [16]. More recently, the work of Conner et al. [17] provided a detailed anatomical and tractography-based reference using the Human Connectome Project for researchers and clinicians alike. Their anatomical and DTI findings are in line with those of Martino et al. [12], as they identified the origins of the IFOF in the inferior occipital gyrus and saw two separate streams, both terminating in areas in the frontal lobe. These studies indicate some variability in the terminations of the dorsal and ventral portions of IFOF, but ultimately, they provide strong evidence to validate the structural differentiation of these tracts.

### 1.3. Functional Framework of the Inferior Frontal Occipital Fasciculus

Due to the structural differentiation that can be seen in the IFOF, there is reason to propose that there might also be a functional differentiation of the dorsal and ventral components of the IFOF. There is still an ongoing debate about the role of the IFOF, especially with respect to the exact functions of the anatomical subcomponents. Current literature reveals three main hypotheses/schools of thought with regard to the functional frameworks of the dorsal and ventral components of the IFOF: (1) the phonological vs. semantic processing hypothesis, (2) the difficult vs. non-difficult task processing hypothesis, and (3) the automatic vs. non-automatic processing hypothesis. Each of these hypotheses is discussed in further detail below. 

### 1.4. Hypothesis #1: Phonological vs. Semantic/Orthographic Processing

The phonological vs. semantic/orthographic processing hypothesis proposes that the dorsal component of the IFOF is associated with tasks like the pronunciation of nonwords that require phonological processing [18,19,20], while the ventral component of the IFOF is associated with tasks like language comprehension that involve semantic and orthographic processing [21,22]. For example, Saur et al. [21] found that behavior associated with repetition of nonwords was related to dorsal tract areas like the superior temporal and frontal premotor regions. Tasks related to auditory comprehension were associated with the middle/inferior temporal regions of the ventral pathway. Klingberg et al. [19] and Steinbrink et al. [20] reported similar findings whereby adults with a history of dyslexia had lower accuracy and reading speed for real words and nonwords, and this behavior was associated with decreased FA in temporal-parietal regions found in the dorsal segment of the IFOF. Ultimately, this hypothesis claims that the functional differentiation of the dorsal and ventral IFOF is dependent on the underlying phonological or semantic processing necessary for a task.

### 1.5. Hypothesis #2: Difficult vs. Non-Difficult Processing

The difficult vs. non-difficult processing hypothesis proposes that the ventral tract is responsible for difficult tasks that have a high processing load while the dorsal tract takes care of more simple tasks. This hypothesis is supported by Lopez-Barroso et al. [23], who found that language learning was elevated in participants with more developed ventral pathway microstructure (i.e., fractional anisotropy). They proposed that the ventral path might provide a supplementary mechanism for learning during high-load conditions when the dorsal stream is overwhelmed. They also propose that this ventral stream might be particularly important when the dorsal stream is still under development [23]. This assertion is supported by Brauer et al. [24] and Brauer et al. [25], who found that, even though children have reduced FA in white matter pathways compared to adults, children predominantly use the ventral aspects of the IFOF pathway (i.e., extreme capsule) during language development, even for tasks that adults would use the dorsal pathway for. Finally, additional support for the difficult vs. non-difficult hypothesis comes from research indicating that higher FA values in the ventral component are associated with increased postoperative fluency in patients who have undergone temporal lobe resections for epilepsy [26,27]. 

### 1.6. Hypothesis #3: Automatic vs. Non-Automatic Processing

The automatic vs. non-automatic processing hypothesis proposes that the ventral and dorsal components of the IFOF are functionally separated into automatic vs. non-automatic processes, respectively. Dávolos et al. [28] conducted an in-vivo tractography study to identify the white matter tracts involved in semantic cognition. They identified a role of the IFOF during tasks involving more automatic patterns of semantic retrieval and less semantic control demands (i.e., when stimuli are congruent). Although they did not discuss a distinction between the dorsal and ventral components of the IFOF, their study shows the role of the IFOF during less controlled semantic decisions [28]. Complementary support for this assertion is noted in Rollans et al. [7], who reported a significant relationship between FA in the overall IFOF and non-automatic tasks (i.e., reading nonwords, serially naming pictures). In their subsequent work, they differentiated the dorsal and ventral portions of the IFOF and found that FA of the dorsal IFOF was associated with tasks involving response selection (i.e., non-automatic), whereas FA of the ventral IFOF was associated with straightforward naming tasks (i.e., automatic) [8]. However, these previous studies did not consider the potential overlap, parallel, and/or crossing fibers associated with the middle and anterior segments of the IFOF. Thus, additional work is needed to confirm these previous findings.

### 1.7. Aims of the Study

The purpose of the current work is to disentangle the three aforementioned hypotheses and provide more specificity with respect to the role(s) of the ventral and dorsal IFOF in language processing. To do this, we need to isolate the left dorsal and ventral fibers at the posterior section of the IFOF (i.e., the initiation points), where the structural differentiation is largest and ‘shared/crossing’ fibers are minimal. Using a series of behavioral tasks that differentially rely on the ascribed dorsal/ventral processes (i.e., phonological vs. semantic, easy vs. difficult, non-automatic vs. automatic), we aim to better understand the functional differentiation of the IFOF in adult readers with a range of reading abilities. Our proposed hypotheses for the roles of the ventral and dorsal IFOF in the phonological-semantic, difficulty, and automaticity processing are outlined in Table 1. 

## 2. Materials and Methods

Participants included adults (N = 32 who were 18+ (Mean Age = 22.87 (4.1)). Inclusion criteria consisted of English as a native or primary language, normal or corrected to normal vision, no contraindications to MRI testing, and age-appropriate scores on non-verbal intelligence testing. Exclusion criteria consisted of a history of hearing impairment, stroke, or neurological disorders (e.g., ADHD). All participants provided written, informed consent and were paid an honorarium of $30 CAD for their participation. This research study was collected as part of a larger research study (see [4] for the fMRI results) and was approved and conducted in accordance with the Research Ethics Board (REB) at the University of Alberta.

The behavioral tasks consisted of ‘awareness’ tasks (phonological [29], orthographic [30,31], morphological [32]) and ‘letter probe’ tasks (phonological, orthographic-phonological, orthographic [33,34]). Participants also completed two standardized measures of reading performance, namely the test of word reading [35].

*Awareness tasks*. The phonological awareness (PA) task contained two elements: (1) a nonword repetition task, whereby participants listened to a nonword then repeated it back, and (2) a phoneme deletion task, whereby they repeated the aforementioned nonword with a target sound excluded. For example, “say daybishocko (without the /sh/)”. The orthographic awareness (OA) task required participants to use their knowledge of orthographically legal letter sequences to make plausibility judgments about which pseudoword was likely to be a real English word. For example, if given ‘qount’ and ‘bount’, participants should select the latter as the correct answer as ‘qount’ violates English spelling rules (i.e., ‘Q’ is always followed by a ‘U’). The morphological awareness (MA) was a sentence-based task whereby participants read incomplete sentences and were required to select the best nonword (from a list of four) that fit the sentence. The four nonwords varied in their morphological suffix. For example, given the sentence ‘Their progress was stopped by an unexpected _____’ and the four options are: postramify, postramic, postramity, postramicize, participants should select ‘postramic’ or ‘postramity’. 

Letter probe tasks. When in the MRI scanner, participants also completed three letter probe tasks. For all tasks, participants heard a word or nonword via headphones and then saw a single letter presented on a screen. Participants decided via a button press (press ‘1’ for yes, press ‘2’ for no) if the aural stimulus contained the visually presented letter. They were asked to respond as quickly and accurately as possible. Three versions of the letter probe tasks were presented: (1) The phonological (P) version that contained nonwords or pseudowords for which participants had no previous knowledge (e.g., the letter ‘N’ in wint). This task required participants to generate the likely spelling of each word and then make a decision about the presence/absence of the letter in the stimulus; (2) The orthographic-phonological (OP) version contained words that had consistent spelling-to-sound correspondences (e.g., the letter ‘A’ in haze). In this case, participants could sound out the word to make a judgment and/or retrieve stored print-based information; (3) The orthographic (O) version included words that had irregular spelling-to-sound correspondences. As such, this task was highly reliant on stored print information as participants had to retrieve the spelling of the word from their lexicon to make accurate real-time judgments. In this latter letter probe task, the visually presented letters could not be identified from sound-based or pronunciation-based information as they were: (a) absent from the pronunciation of the word (e.g., the letter ‘c’ in yacht); (b) ambiguous with respect to the associated phonemes (e.g., the letter ‘C’ in cello); or (c) highly associated with a specific phoneme (e.g., the letter ‘G’, pronounced /g/, in the word got), but pronounced differently in the presented stimulus (e.g., the letter ‘G’ in regime).

The letter probe tasks were programmed and presented via EPrime software (Psychology Software Tools, Inc., Pittsburgh, PA, USA, Version 2.0, http://www.pstnet.com). The audio files for each condition were preprocessed and calibrated for frequency using the Audacity software. Important stimulus characteristics were controlled for every condition (i.e., onset time of stimulus, stimulus duration). Each letter probe condition (i.e., P, OP, and O) contained a total number of 75 words/nonwords, and the stimuli were matched on the following characteristics across and within the tasks: frequency of word, orthographic and phonological neighborhood size, number of phonemes, syllables and morphemes, word length, summed bigram frequency and summer bigram frequency by position [36]. Task order and word order within each task were randomized for each participant. The outcomes from these spelling tasks are mean response time to correct trials and mean accuracy.

### 2.1. DTI Collection

Brain images were acquired on a 3 T Siemens MAGNETOM Prisma scanner with all images positioned along the anterior-posterior-commissure line. Diffusion-weighted images were collected using a dual-spin-echo single-shot echo-planar imaging sequence. The sequence included thirty non-collinear directions of diffusion-sensitizing gradients, with a b-value = 1000 s/mm^2^, TR = 4910 ms, and echo time = 164 ms. Axial slices (N = 94; slice thickness = 1.5 mm; voxel size = 0.7 × 0.7 × 1.5 mm) were obtained. The image matrix was 128 × 128 with 75% phase partial Fourier zero-filled to 256 × 256. Diffusion image acquisition took approximately 8 min. For registration purposes, anatomical scans were also collected using a high-resolution axial T1 MPRAGE sequence: repetition time (TR) = 1700 ms, echo time (TE) = 2.21 ms, number of slices = 176, base resolution 256 × 256 × 176 with a voxel size of 1 × 1 × 1 mm, scan time of 4.50 min.

### 2.2. DTI Analysis

Diffusion scans were analyzed using Explore DTI Version 4.8.6 [37]. First, raw images were visually inspected for motion artifacts, which included assessments of gross subject motion (seen as rings or waves), signal artifacts (hyperintensities, etc.), signal dropouts (black spots), or missing data files. Next, preprocessing of the diffusion data was implemented. This included corrections for signal drift (quadratic), Gibbs ringing (5 non-DWIs; Lamda = 100; iterations = 100; step size = 0.01), registration between diffusion images and structural images (i.e., normalization), masking to remove non-diffusion weighted signal (kernel = 9; 0.5 for non-DWIs; 0.8 for DWIs), distortion correction for non-rigid EPI distortions, and corrections for individual subject motion.

**Figure 1:**
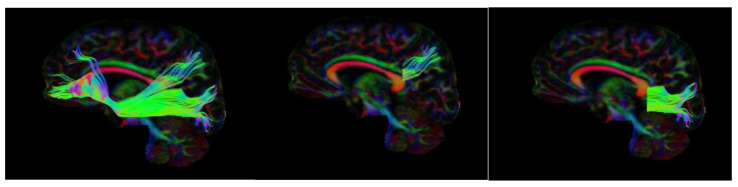
Representative isolation of the left IFOF tract for one participant. Left = Entire IFOF; Middle = Dorsal segment of the IFOF; Right = Ventral segment of the IFOF.

Tractography. A tensor model was applied, and voxelwise maps associated with fractional anisotropy were generated and used to complete tractography analysis. Left lateralized regions of interest were drawn based on a template following the guidelines provided by Wakana et al. [38] and warped into the space of each individual brain. A minimum FA threshold of 0.20 was used to initiate and continue tracking, and a maximum turning angle of 30 degrees was applied as a cutoff to stop tracking. A step length of 1 mm was used in between each calculation (min = 1 mm and max = 500 mm). The first ROI was between the posterior edge of the parieto-occipital sulcus and the posterior edge of the cingulum, while the second ROI was at the anterior edge of the genu [38]. After the IFOF was isolated, it was separated into the dorsal and ventral regions following the tractography protocol created by Rollans et al. [8]. ROIs for the posterior segments of the ventral and dorsal tracts were selected based on the visual distinction between the tracts that could be seen after the entire IFOF was isolated. The ROIs differed between each participant, but ultimately, the tracts were separated at their posterior coronal cross-sections, where the dorsal tract began to trend upwards (see Appendix A for individual tract visualization). Representative images for the total IFOF, as well as the dorsal and ventral segments, can be found in Figure 1.

### 2.3. Statistical Analysis

SPSS Statistics were used to complete the statistical analysis [39]. Means and standard deviations associated with the letter probe tasks (i.e., phonological, orthographic-phonological, orthographic) and accuracy rates from the awareness tasks (i.e., phonological, orthographic, morphological) were extracted (Table 2). In addition, means and standard deviations associated with FA and MD values from the dorsal IFOF and ventral IFOF were obtained (Table 3). To address our hypotheses, a series of regression analyses were run with the FA (and MD) values of the ventral and dorsal components of the IFOF as the criterion and the awareness tasks or letter probe tasks as the predictors. This served to control for shared variance among the behavioral tasks, which allowed us to assess the unique contributions of each process to the IFOF segments. Standardized regression coefficients and associated *p*-values are reported. In instances where no IFOF component could be isolated, the missing data were removed on a case-by-case basis. In some cases, behavioral data were missing, and a mean group substitution was employed.

## 3. Results

Participants (N = 23 females) had a range of reading abilities with standardized scores on the Test of Word Reading Efficiency (TOWRE) from 68–113 for Real Word Fluency and 65–120 for Nonword Fluency [36]. The mean years of schooling was = 16.4 (2.6). The mean behavioral measures are reported in Table 2. The mean diffusion measures are reported in Table 3. The diffusion measures associated with the dorsal and ventral segments of the IFOF were significantly related (Supplementary Figure S1). Specifically, the FA of the dorsal and ventral segments was significantly correlated, r = 0.410, *p* = 0.020, as was the MD of the dorsal and ventral segments, r = 0.713, *p* < 0.001. The scatterplots depicting the relationships between dorsal, ventral, and total IFOF FA/MD and the awareness and letter probe tasks can be found in Supplementary Figure S2.

### 3.1. Dorsal IFOF

**Figure 2:**
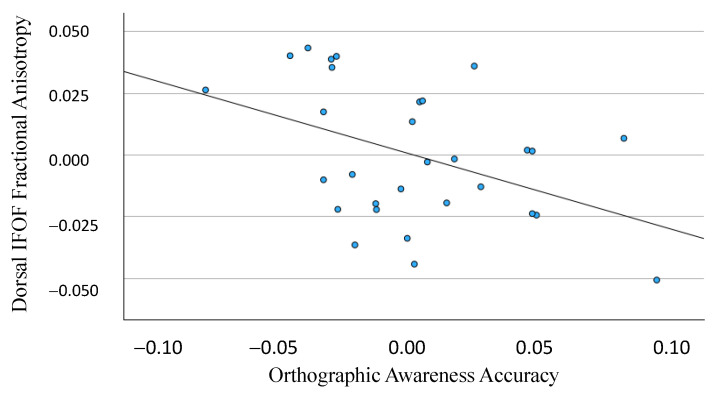
Partial regression plot depicting the relationship between dorsal IFOF FA and orthographic awareness accuracy. *β* = −0.436, *p* = 0.023.

The FA of the dorsal segment was significantly related to the orthographic awareness accuracy, *β* = −0.436, *p* = 0.023. No other relationships with behavior were found for either FA or MD (see Figure 2).

### 3.2. Ventral IFOF

**Figure 3:**
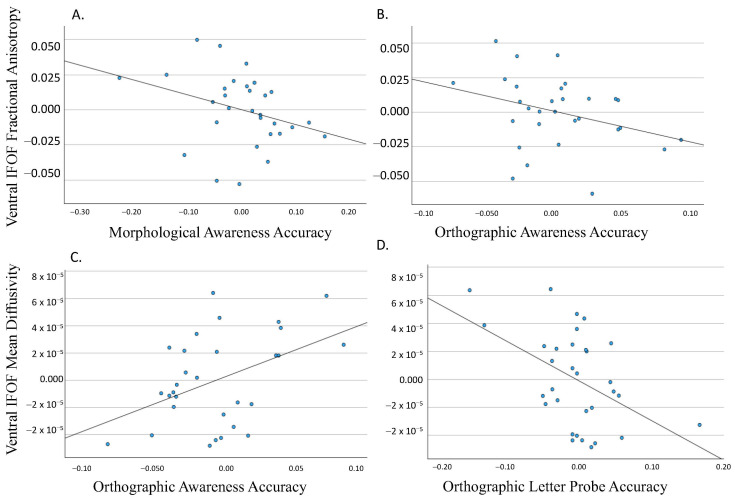
Partial regression plot depicting the relationship between (1) ventral IFOF FA and morphological awareness ((**A**); *β* = −0.406, *p* = 0.095) and orthographic awareness ((**B**); *β* = −0.328, *p* = 0.090) and (2) ventral IFOF MD and orthographic awareness ((**C**); *β* = 0.468, *p* = 0.018) and orthographic letter probe accuracy ((**D**); *β* = −0.657, *p* = 0.009).

The FA of the ventral segment approached significance for morphological awareness, *β* = −0.406, *p* = 0.095, and orthographic awareness, *β* = −0.328, *p* = 0.090 (Figure 3A,B). The MD of the ventral segment was significantly related to orthographic awareness, *β* = 0.468, *p* = 0.018 (Figure 3C). The MD of the ventral segment was also significantly related to orthographic accuracy from the letter probe task, *β* = −0.657, *p* = 0.009 (Figure 3D). A summary of the significant findings can be found in Table 4.

## 4. Discussion

Here, we set out to better specify the role of the IFOF in language processing. Given the three reported hypotheses in the literature, we systematically explored behavior-FA/MD relationships within the dorsal and ventral segments of the IFOF in skilled and impaired readers. Interestingly, we found predominantly ventral associations with the various behavioral tasks that were associated with orthographic processing. We found no relationships with any of the phonological-based tasks. Together, these findings do not support hypotheses that functionally separate the dorsal and ventral segments of the IFOF. Instead, our findings are in line with the notion that the IFOF functions primarily as a ventral language tract, with strong connections to orthographic processes and retrieval of familiar semantic units.

### 4.1. Functional Specificity of IFOF Segments

Although there is evidence for the structural differentiation of the dorsal/ventral IFOF [13,14,15,16], the functional specificity of these dorsal/ventral segments has remained ambiguous. Using the three predominant hypotheses for dorsal/ventral IFOF language contribution, we set out to provide specificity on the functional roles of the dorsal and ventral IFOF. Ultimately, we found consistent relationships between the ventral IFOF and orthographic processing tasks but no relationships with the phonological-based tasks. As such, our findings do not line up directly with any of the outlined hypotheses. An alternative hypothesis could be argued, namely, that the IFOF is strictly involved in the retrieval of semantic rules from storage (i.e., task demands strongly linked to the Orthographic Awareness and Orthographic letter probe tasks). In this case, the lack of findings with the remaining behavioral tasks, which could utilize differential processing, would be reasonable. Ultimately, much more work is needed to explore this ‘new’ hypothesis fully, and we caution the reader to avoid over-generalizing the findings reported in the current study as null effects are difficult to ‘prove’ if you will.

Two major distinctions between our work and previous work exploring the dorsal/ventral segments of the IFOF [7,8,24,25] include our isolation approach that focused on the most posterior segments of the IFOF and that we used an adult population with a range of reading abilities. With respect to the former, we opted for a clear structural separation between the dorsal and ventral projections as the middle and anterior portions of the dorsal/ventral IFOF tract may share many crossing and parallel fibers that ultimately make it difficult to disentangle the tract-behavior relationships. By focusing on the initiation projections in the parietal and temporal regions, we hoped to minimize the potentially confounding effects of shared fibers. With respect to the latter, reading ability/disability studies are predominantly conducted with children [2,19,24,25,40,41] as such information is important for our understanding of language development and potential loci of breakdown. However, we cannot generalize the findings from child-based samples to adult populations, as reading networks and pathways are continuously modified in response to reading refinement, skill development, maintenance, and potential compensatory mechanisms in the case of reading impairment. The results from this work provide converging evidence for the primary role of the IFOF in orthographic processing for adults.

What is somewhat surprising is the lack of findings associated with the dorsal IFOF. Regardless of which hypothesis was supported, we expected to see some tasks associated with the dorsal IFOF. There is much evidence that the dorsal pathways of the brain are associated with phonological-based processing [41,42], yet we did not find any relationships with the phonological tasks used in the current work. One may argue that our limited findings associated with the dorsal IFOF were a result of our tractography approach, namely the isolation of the unique sections of the tract in the posterior brain areas. For example, an examination of the termination points of the IFOF, instead of the initiation points as conducted in this study, may yield different results. Sarubbo et al. [16] provided detailed information regarding the superficial and deep layers of the IFOF, with the former being comprised of upwardly directed fibers that terminated in the inferior frontal gyrus and the latter being comprised of posterior, middle, and anterior fibers that primarily projected to the middle frontal gyrus. Given the extensive imaging work that describes the differential function of the inferior and middle temporal gyri, it would be reasonable to hypothesize that the superficial and deep layers of the IFOF may correlate differentially with language function [18]. However, even when we explored the entire IFOF (see supplementary material), we did not find any relationships with the phonological awareness task or the phonological letter probe task. Such null effects, in conjunction with the multiple relationships found for the ventral segment of the IFOF, compel us to propose that the IFOF should be considered a ventral language tract in the adult brain.

Finally, language is a highly complex, intricate, and dynamic process that involves several areas of the brain [6]. It might be the case that the three proposed theories to explain the roles of the IFOF are oversimplifying the process. To fully understand the functional distinction of the IFOF, a larger, big-picture examination of the connectivity between different brain regions, which should include functional connectivity as well as structural connectivity, might be more useful than observations of isolated and static tracts alone. The IFOF is part of a larger language network, so it might be fruitful to look at greater network connections; consequently, the correlations observed in our study should be viewed with caution.

### 4.2. Limitations of the Study

The absence of data for a few participants served as a major limitation of this study. In some instances, the IFOF tract could not be sufficiently isolated, and the data were excluded on a case-by-case basis. In addition, the unequal representation of males/females and left/right-handed participants precluded any analyses with respect to these potential factors. Ultimately, the fiber tract isolation itself introduced human error, which is another limitation. Protocols written by Wakana et al. [38] were useful in guiding the drawing of ROIs, but in practice, some regions differed from the literature. These limitations during tractography could have some effects on the diffusion measures and, subsequently, the correlations that were obtained. Additionally, this study focuses on diffusivity measures like FA, and some consideration is given to MD as well. Including other diffusion measures, like axial or radial diffusivity, may be useful in the future (albeit at the added expense of additional correlations and inflation of Type 1 error) as they provide different information about white matter structure. A similar limitation is observed in regard to the behavioral measures. Specifically, we only included accuracy-based tasks in the current study to maintain some uniformity in variable measurement. Reaction time variables provide a unique perspective on the IFOF’s involvement in language processing efficiency [5]. The possible relationship between reaction time and the diffusivity measures could provide more clarity on the role of the ventral and dorsal IFOF during those behavioral tasks. Additionally, a larger sample size of participants would help minimize Type 1 errors. Ultimately, we chose one approach out of many that can be used to study the IFOF. Future studies should also keep in mind the complexity of the language networks; consequently, they might want to study the connectivity of these regions instead of focusing on single tracts. This would help deepen our knowledge about the role of the IFOF in reading and language processing. 

## 5. Conclusions

Ultimately, the goal of this study was to determine which of the three proposed hypotheses best explains the functional roles of the dorsal and ventral components of the IFOF. According to the consistent correlations between the ventral IFOF and accuracy for the orthographic awareness task, our findings support the notion that the IFOF supports access to familiar semantic units and operates as a ventral language tract in adults. Nevertheless, these findings must be interpreted with caution due to the lack of correlations observed with other behavioral tasks, including phonological tasks. Future work aiming to elucidate the functional distinctions between component tracts like the IFOF, might want to focus on the connections between different brain areas instead of just observing a singular tract by itself.

## Figures and Tables

**Table 1:** Expected findings that would support the three current theories about the functional distinction of the IFOF. If the selected hypothesis is correct, the behavioral measures outlined should correlate with the FA or MD measures of the appropriate dorsal and ventral segments of the IFOF. O = Orthographic spelling task, OP = Orthographic-Phonological spelling task, P = Phonological spelling task, OA = Orthographic awareness, PA = Phonological awareness, MA = Morphological awareness.

	Dorsal-IFOF	Ventral-IFOF
Phonological-semantic hypothesis	P, OP, PA	O, OA, MA
Difficulty hypothesis	P, MA	O, OP, PA, OA
Automaticity hypothesis	P, OA, PA, MA	O, OP

**Table 2:** The mean behavioral accuracy (standard deviation) for each task.

Awareness Tasks			Letter Probe Tasks		
Phonological	Orthographic	Morphological	Phonological	Phonological-Orthographic	Orthographic
73.0 (18.1)	89.5 (4.5)	90.7 (13.4)	85.9 (4.5)	84.9 (6.0)	76.0 (8.1)

**Table 3:** The mean diffusion measures (standard deviation) for the total, dorsal, and ventral IFOF.

Total IFOF FA	Total IFOF MD	Dorsal FA	Dorsal MD	Ventral FA	Ventral MD
0.48 (0.02)	0.0008 (0.00002)	0.45 (0.03)	0.0008 (0.00003)	0.51 (0.03)	0.0008 (0.00003)

**Table 4:** Summary of the significant coefficients for each of the regression models. * *p* < 0.05 (two-tailed); ^+^ *p* < 0.05 (one-tailed).

	Dorsal-IFOF	Ventral-IFOF
Phonological-semantic hypothesis		O *, OA *, MA ^+^
Difficulty hypothesis		O *, OA *
Automaticity hypothesis	OA *	O *

**Figure A1:**
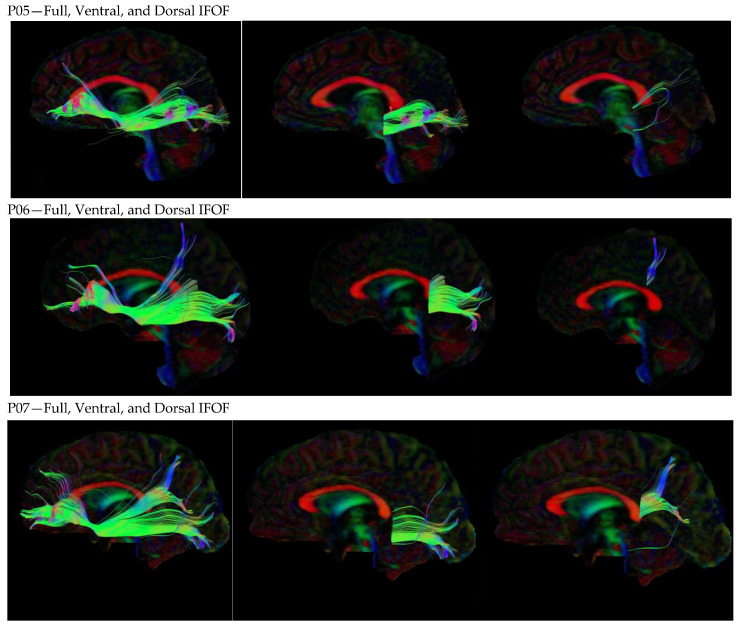

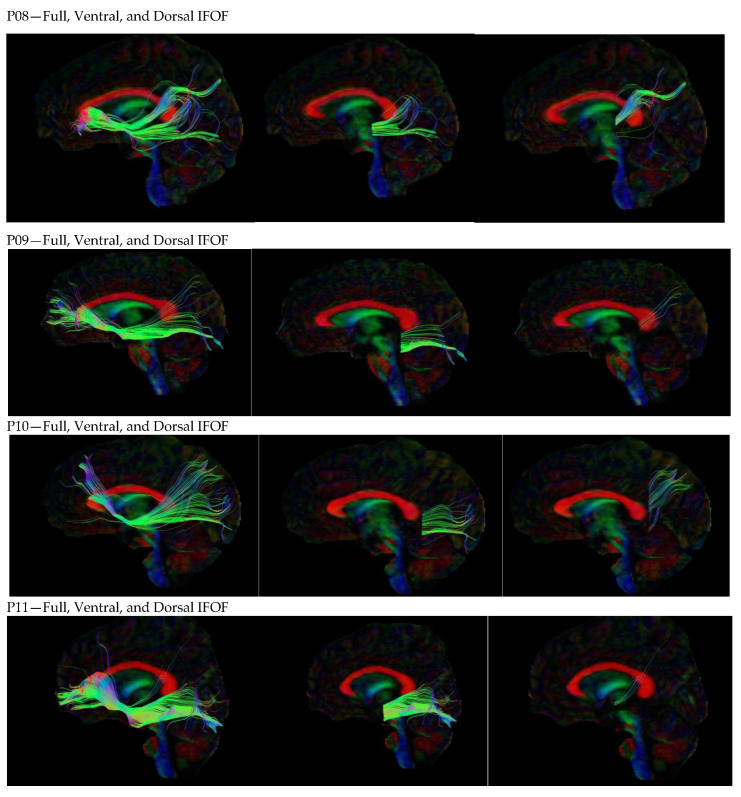

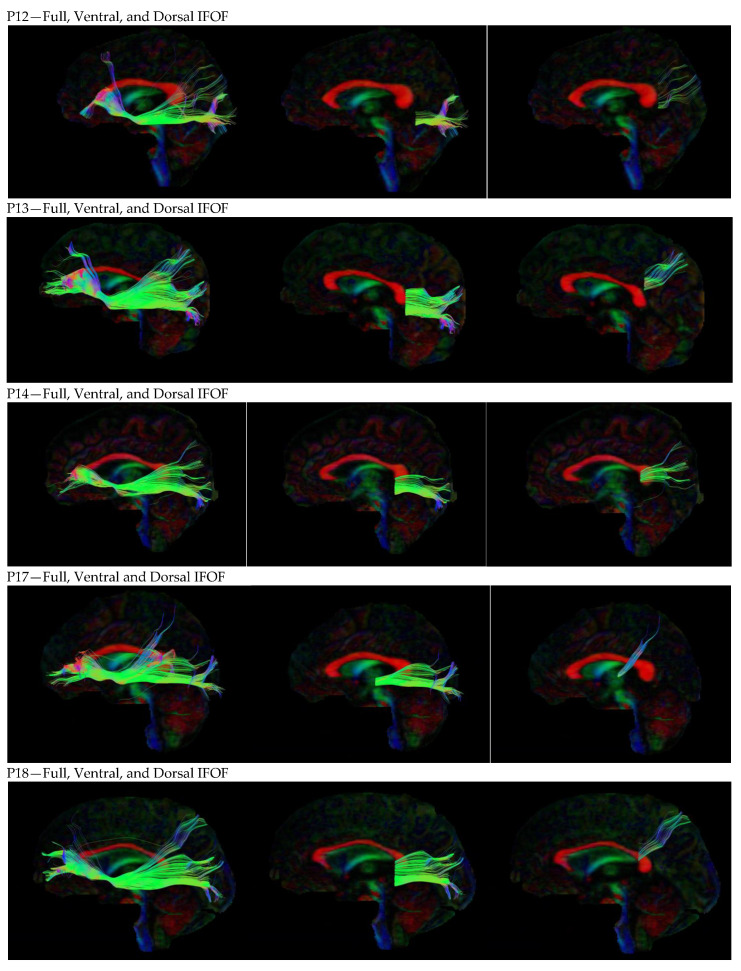

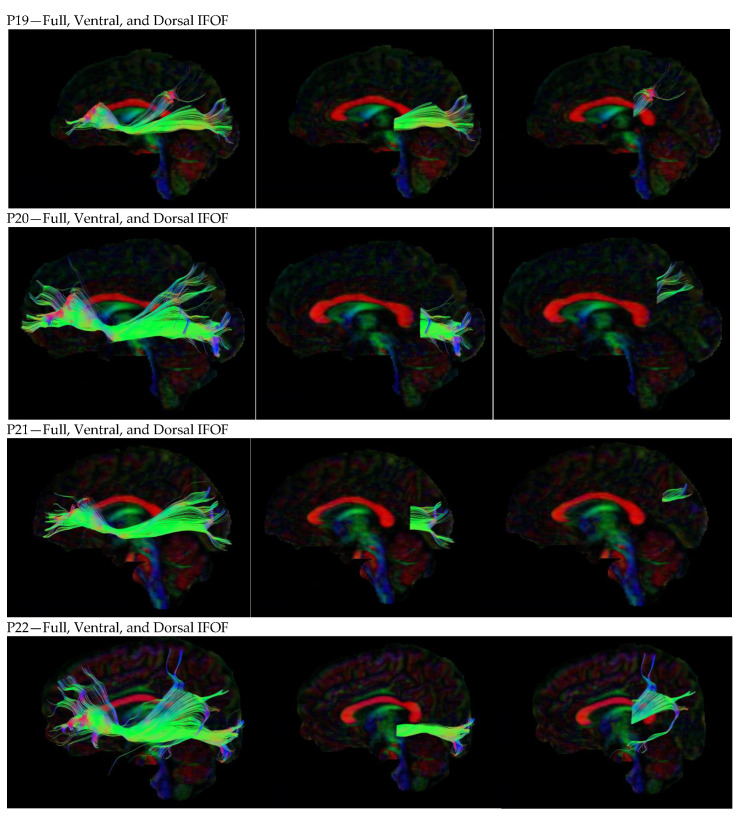

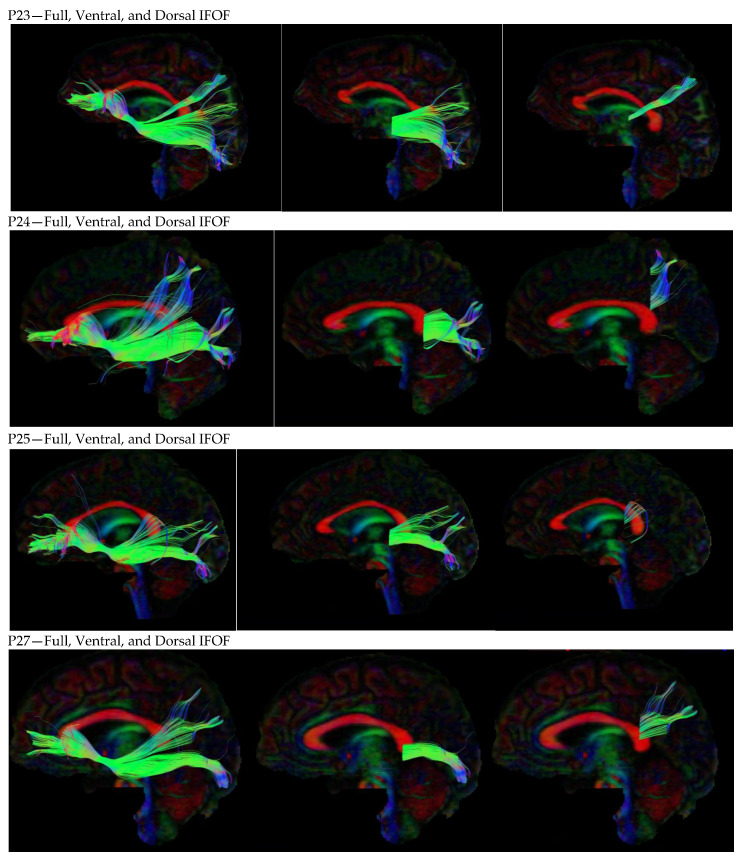

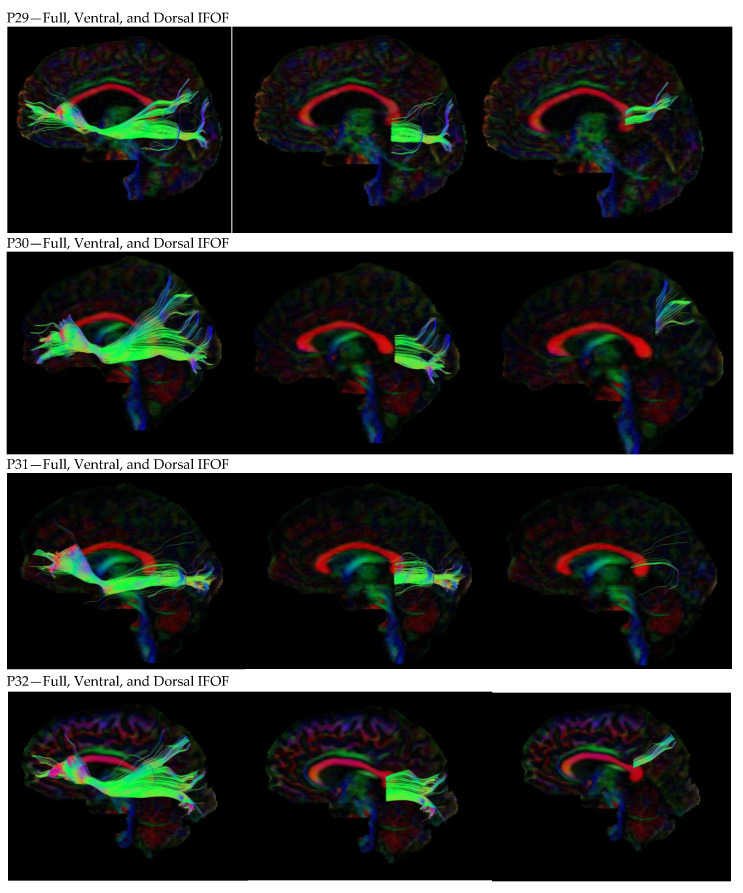

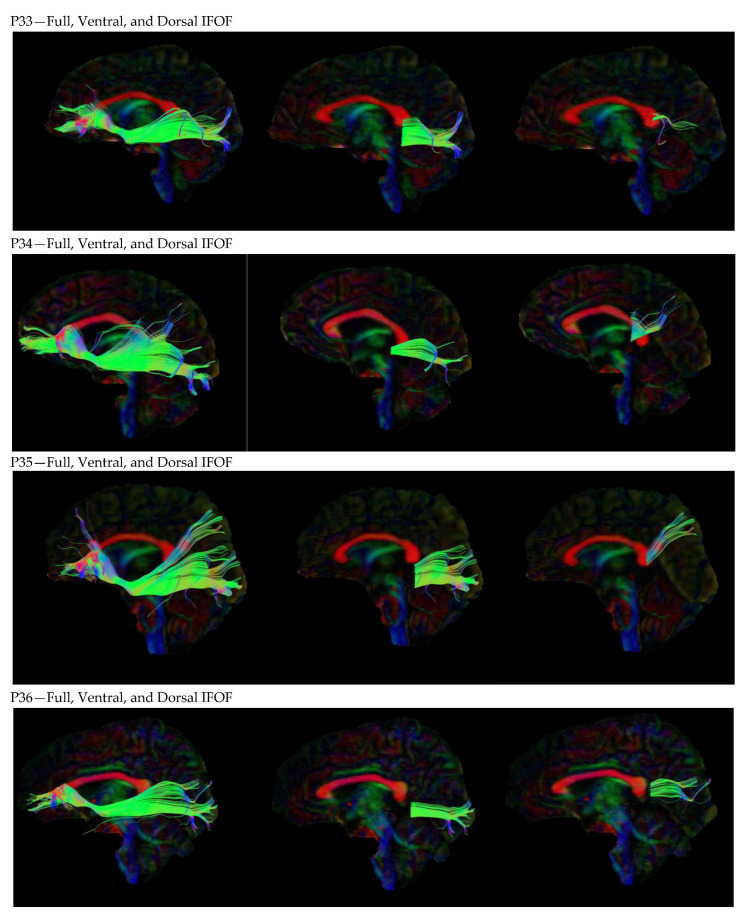

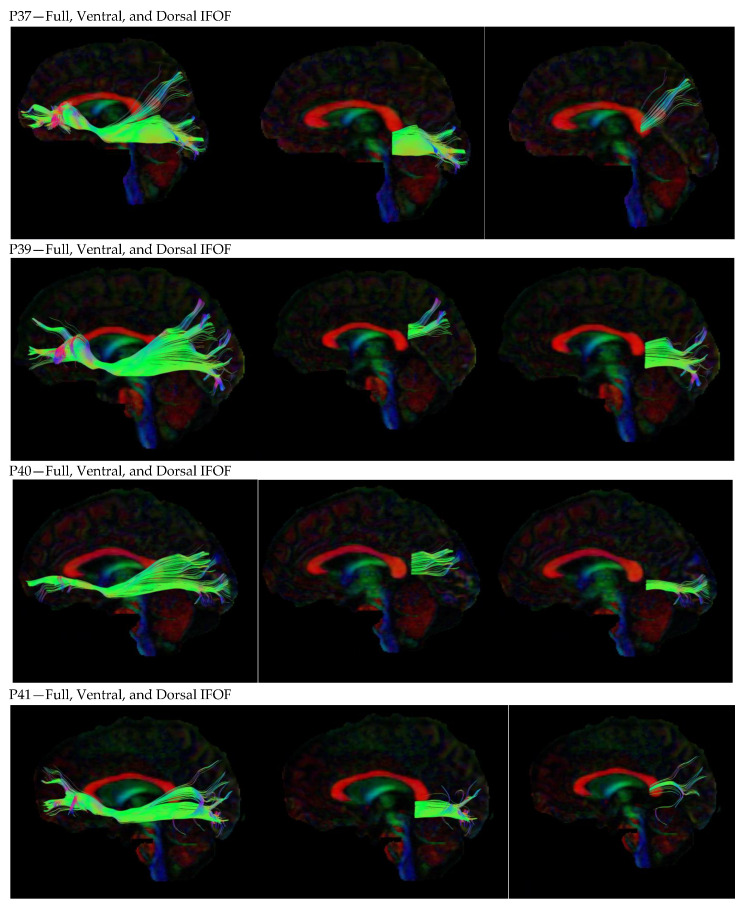
Individual Segmentation Images for Each Participant (N = 32).

## Data Availability

Data are available on request due to restrictions of the ethical review board.

## Supplementary Materials

The following supporting information can be downloaded at: https://www.mdpi.com/article/10.3390/neurosci5010003/s1, Figure S1: Scatterplot representing the relationship between fractional anisotropy (Left plot; Spearman’s r = 0.410, *p* = 0.02) and mean diffusivity (Right plot; Spearman’s r = 0.713, *p* < 0.001) in the dorsal and ventral segments of the IFOF. Figure S2: Scatterplots depicting the simple correlation between Dorsal (A,B), Ventral (C,D), and Total IFOF (E,F) and reading behaviour. Red boxes indicate significant correlations (*p* < 0.05, one-tailed).
