# Supplementary material for: Moving towards an Understanding of the Role of the Inferior Fronto-Occipital Fasciculus in Language Processing
Source: NeuroSci. 2024 Jan 2;5(1):39–58. doi: 10.3390/neurosci5010003 (PMC11523719; doi:10.3390/neurosci5010003)
Supplement: Supplementary file 1 [file neurosci-05-00003-s001.zip › neurosci-2776354-supplementary.pdf]

NeuroSci

MDPI

Supplementary Material

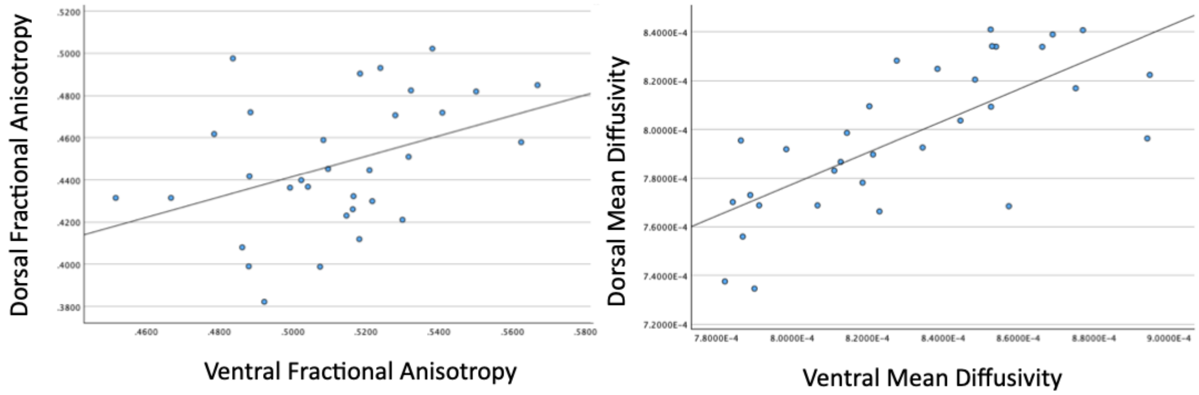

Figure S1. Scatterplot representing the relationship between fractional anisotropy (Left plot; Spearman's r = .410, $p = 0.02$) and mean diffusivity (Right plot; Spearman's r = .713, $p < 0.001$) in the dorsal and ventral segments of the IFOF.

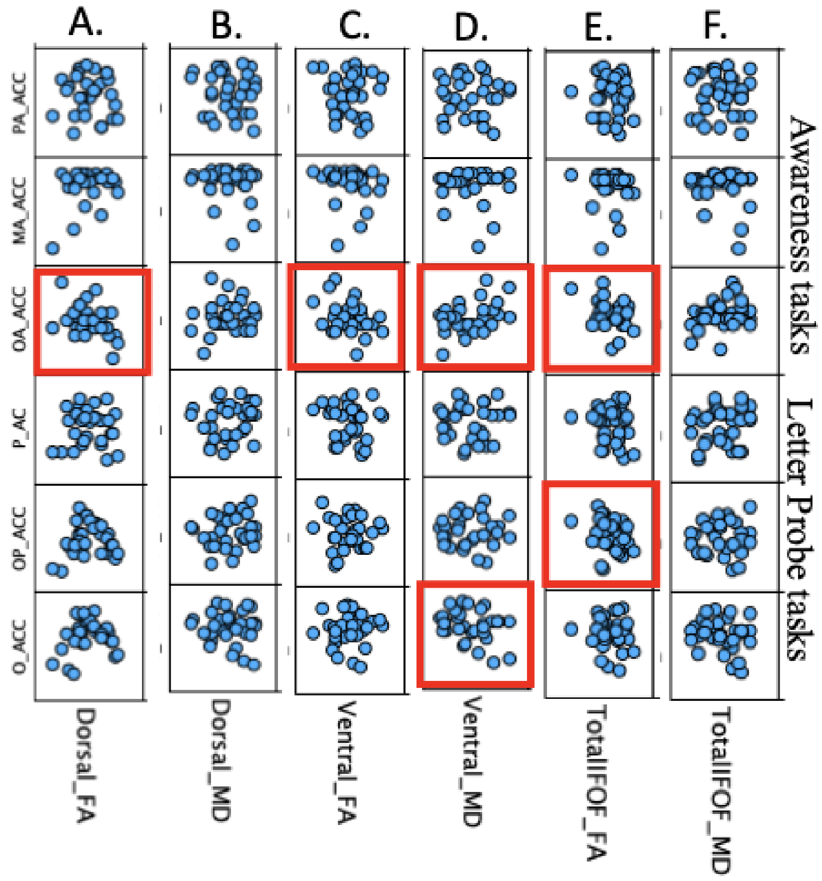

Figure S2. Scatterplots depicting the simple correlation between Dorsal (A & B), Ventral (C & D), and Total IFOF (E & F) and reading behaviour. Red boxes indicate significant correlations (p<0.05, one-tailed).
